# Role of Transcranial Doppler in the Evaluation of Vasculopathy in Tuberculous Meningitis

**DOI:** 10.1371/journal.pone.0164266

**Published:** 2016-10-10

**Authors:** Mei-Ling Sharon Tai, Vijay K. Sharma

**Affiliations:** 1 Division of Neurology, Department of Medicine, Faculty of Medicine, University Malaya, Kuala Lumpur, Malaysia; 2 Division of Neurology, National University Health System, Singapore, Singapore; Medical University Innsbruck, AUSTRIA

## Abstract

**Background:**

Vascular complications are important causes of cerebral infarction in tuberculous meningitis (TBM).Transcranial Doppler ultrasonography (TCD) is a non-invasive tool that can provide real-time information about cerebral hemodynamics. However, the literature on the role of TCD in the diagnosis or monitoring of vasculopathy associated with TBM is scarce. We explored the role of TCD in the diagnosis and monitoring of TBM-related vasculopathy of the major intracranial arteries.

**Methods:**

Consecutive patients with TBM admitted to our tertiary center between 2011 and 2015 were included. All patients underwent TCD evaluation within 2 weeks of hospitalisation and it was repeated 2 weeks later. Mean flow velocity (Vmean) and pulsatility index (PI) were recorded. Flow velocities obtained from the submandibular internal carotid artery were also measured to calculate the Lindegaard ratio. A correlation was made between the patients who demonstrated vasculopathy on TCD, and patients with confirmed focal narrowing on computed tomography angiography (CTA) or magnetic resonance angiography (MRA). The modified Rankin scale (mRS) was used to assess the clinical outcome at three and six months.

**Results:**

A total of 36 patients were recruited. Focally elevated flow velocities in the middle cerebral artery (MCA) were observed in 11 (30.6%) patients, bilaterally in 6 of them. The Lindegaard ratio was elevated (>3) in 10 (90.9%) of them, which occurred as early as the fourth day of hospitalization and persisted as long as four months. Eighty percent of patients with TBM vasculopathy by TCD criteria, also had narrowing on CTA or MRA. Ten patients (27.8%) achieved good outcome (mRS 0–2) at 3 months, which increased to 13 (36.1%) at 6 months.

**Conclusion:**

A considerable proportion of patients with TBM develops intracranial vasculopathy, which can be reliably diagnosed and monitored using TCD.

## Introduction

Tuberculosis (TB) is a leading cause of morbidity and mortality. [1, 2] It can involve the central nervous system as primary or secondary infection of the meninges (tuberculous meningitis, TBM) or brain parenchyma. [3] According to data from the World Health Organization, 40,000 people suffered from TB in Malaysia in 2014 of which 2400 (6%) died. [3] Extrapulmonary TB was diagnosed in 3,055 cases (7.6%). [3]

Cerebral infarction is a recognized complication of TBM. [1, 2] Vascular complications are often responsible for cerebral infarction and for neurological sequelae among adult survivors of TBM. [2] The literature on the use of transcranial Doppler (TCD) for the evaluation of vascular complications in TBM is scarce. Although TCD has been used in the diagnosis and monitoring of TBM-related vasculopathy in children [4, 5], we could not find any publication describing its use in adult patients.

We hypothesized that the pattern of vasculopathy due to TBM would be similar to vasospasm in patients with subarachnoid hemorrhage (SAH), in the form of elevated flow velocities in the affected intracranial arteries and a raised Lindegaard ratio. We aimed at exploring the role of TCD in the diagnosis and monitoring of TBM-related vasculopathy in the major intracranial arteries.

## Methodology

This was a cohort study of patients with TBM with prospective follow-up and prospective inclusion of new cases.

### (a) Patient selection

The study was conducted at the University Malaya Medical Center (UMMC), a tertiary hospital in Kuala Lumpur, Malaysia between 2011 and 2015. The study was approved by the institutional ethics committee of University Malaya Medical Center (890.27). All patients or their legally acceptable representatives provided written informed consent for the study. All patients with TB meningitis admitted during the study period were included.

TBM was diagnosed using appropriate clinical features, abnormal cerebrospinal fluid (CSF) biochemical profile and demonstration of acid-fast bacilli (AFB) on direct smear, culture, polymerase chain reaction (PCR) or biopsy. [6] TBM was classified as “definite” if cerebrospinal fluid (CSF) acid-fast bacilli (AFB) direct smear/mycobacterial culture/polymerase chain reaction (PCR) for mycobacteria tuberculosis were positive. [6] In addition, acid-fast bacilli (AFB) seen in the context of histopathological changes consistent with tuberculosis (TB) in the brain or spinal cord, together with symptoms, signs and CSF changes suggestive of TB, or visible meningitis (on autopsy) were also criteria for definite TBM. [6]

TBM was defined as “probable” if the patients fulfilled the clinical entry criteria together with a total diagnostic score of ≥10 points (when cerebral imaging was not available) or ≥12 points (when cerebral imaging was available) plus exclusion of alternative diagnoses. At least 2 points came from CSF or cerebral imaging criteria. [6] TBM was termed as “possible” when the patients fulfilled the clinical entry criteria and total diagnostic score of 6–9 points (when cerebral imaging was not available) or 6–11 points (when cerebral imaging was available) plus exclusion of alternative diagnoses.

The severity of TBM at the time of hospitalization was divided into three stages, according to British Medical Research Council criteria. [7] The patients in stage 1 were fully conscious and rational (Glasgow Coma Scale; GCS 15), with meningeal signs but no focal neurological deficit. [7] The patients in stage 2 had GCS score 11–14 or 15 with focal neurological signs. [7] The patients in stage 3 had a GCS score of 10 and less. [7]

All patients were treated with intensive and maintenance antituberculous therapy for 12–18 months according to the British Infection Society guidelines. [8] The TBM patients who died primarily due to causes other than neurological conditions, and immunocompromised patients who died from their primary disease were excluded.

### (b) Demography, clinical and imaging characteristics

We collected information on demographic characteristics, clinical features at the time of hospitalization, neuroimaging findings, treatment, clinical progress and outcome. Lumbar puncture was performed on admission. CSF opening pressure was recorded. In addition, CSF was sent for biochemistry, FEME, AFB direct smear, AFB culture and TB polymerase chain reaction (PCR). Lumbar puncture was repeated in some patients who developed clinical deterioration.

All the patients had computed tomography (CT) scan of brain on admission to the hospital.

Serial CT scan of the brain with computed tomography angiography (CTA) or magnetic resonance imaging (MRI) and angiography (MRA) of the brain were performed during hospitalization. Repeat CT scan and/or MRI of the brain were performed when there was a change in the clinical status.

The presence, location and severity of cerebral infarction and intracranial arterial narrowing were documented. In addition, the presence of other neuroradiological changes such as hydrocephalus was recorded. The patients with hydrocephalus had insertion of external ventricular drainage (EVD) and/or ventriculoperitoneal (VP) shunt.

### (c) TCD

All TCD evaluations were performed using a 2-MHz TCD machine (Sonara, Viasys).

All the patients were evaluated with Transcranial Doppler (TCD) within 2 weeks of hospitalization and 2 weeks later. Additional TCD evaluations were performed in some patients if there was a clinical deterioration. Some patients had further TCD evaluation at 3 and 6 months.

The systolic, diastolic and mean blood flow velocities (Vmean) of major intracranial arteries were measured for various intracranial arteries, insonated via transtemporal, transorbital and transforaminal windows. Vmean was calculated as: (Peak systolic velocity-end diastolic velocity)/3 + end diastolic velocity. Vmean of 60–90 cm/s represented the normal range for the middle cerebral artery (MCA). [4, 9] In patients with hydrocephalus, TCD was performed after the insertion of external ventricular drainage (EVD).

The pulsatility index (PI) was obtained for each intracranial artery as (Peak systolic velocity-end diastolic velocity)/mean flow velocity. [4, 5, 10, 11] A value of 0.6–1.1 represented the normal range for the PI in the intracranial arteries. [4, 5, 10, 11, 12]

There are three phases of TBM-related vasculopathy as determined by TCD. In phase I vasculopathy, TCD shows an increase of Vmean and normal to moderately reduced PI. In Phase II, the Vmean is reduced and PI is decreased. In Phase III, there is nearly complete absence of blood flow in at least one basal artery. The PI is very low and can approach 0. Patients at phase III have Vmean <40 cm/s resulting in severe residual neurological deficit or death. [4] An evaluation of the three TCD phases was made in all of the TBM patients.

The criteria used for the diagnosis of MCA vasculopathy were as follows [13, 14]:

1. Vmean >120 cm/s in the affected MCA
2. peak systolic flow velocity (PSV) >157 cm/s in the affected MCA
3. side-to-side difference of mean velocity in the MCAs >30 cm/s.

Furthermore, a Vmean >100 cm/s in the affected anterior cerebral artery (ACA), Vmean >95 cm/s in the basilar artery (BA), and Vmean >85 cm/s in the affected posterior cerebral artery (PCA), indicated vasculopathy in the respective vessels. [13, 14] Vmean >85 cm/s in the vertebral artery and Vmean >100 cm/s in the internal carotid artery siphon represented TBM related vasculopathy.

The Lindegaard ratio was calculated as the ratio of the MCA Vmean to the extracranial internal carotid artery Vmean (MCA/ICA ratio). [11] An assessment of whether the patients developed Lindegaard ratio of more than three and more than six, was made. Severe vasculopathy was defined as Lindegaard ratio of more than six.

### (d) Outcome measures

Modified Rankin scale (mRS) was used to assess the clinical outcome at three and six months. The grading of mRS is as follow:

- 0—No symptoms.
- 1—No significant disability. Able to carry out all of his usual activities, despite some symptoms.
- 2—Slight disability. Able to look after his own affairs without assistance, but is unable to carry out all of his previous activities.
- 3—Moderate disability. Requires some help, but is able to walk unassisted.
- 4—Moderately severe disability. Unable to attend to his own bodily needs without assistance, and is unable to walk unassisted.
- 5—Severe disability. Requires constant nursing care and attention, is bedridden and incontinent.
- 6—Death. [7]

Good clinical outcome was defined as mRS of 0–2, and poor outcome was defined as mRS of 3–6.

### (e) Statistical analysis

The Vmean and PI between patients admitted with Stage 1 and advanced stages (stage 2 and 3), were compared. In addition, the Vmean and PI between patients who had good outcome (mRS 0–2) and poor outcome (mRS 3–6) at 3 and 6 months were compared. A correlation was made between the patients who had vasculopathy based on TCD criteria and arterial narrowing on CTA or MRA.

All descriptive statistics were analyzed using Statistical Package for Social Sciences, SPSS (Version 20.0, SPSS Inc., Chicago, USA). Results were recorded as frequencies, means± standard deviation and p values. Continuous variables (Vmean and PI) were expressed as means ± standard deviation. Continuous variables (Vmean and PI) between the two groups were compared by using Student’s t test. Pearson correlation was performed to assess the correlation between lumbar CSF opening pressure and the pulsatility index (PI) of MCA (insonated within 2 weeks of hospitalization). A p value of < 0.05 (two-tailed p value) was defined as statistical significant.

## Results

### Demography and baseline characteristics of TBM patients

A total of 36 patients were recruited. Baseline characteristics are presented in Table 1. Briefly, mean age was 38.28 years (range 16–75 years) with slight male (55.6%) predominance. Majority (63.9%) of the patients presented with stage 2 TBM and three (8.3%) patients were HIV-infected.

**Table 1:** Demography and baseline characteristics of TBM patients.

	Patients, n = 36
**Age** (mean±SD)	38.28±12.83 (range 16–75, median 35)
**Gender** (n, %)	
Male	20 (55.6%)
Female	16 (44.4%)
**Ethnic group** (n, %)	
Malay	10 (27.8%)
Chinese	6 (16.7%)
Indian	13 (36.1%)
Non-Malaysians	7 (19.5%)
**Stage of illness on admission** (n, %)	
Stage 1	6 (16.7%)
Stage 2	23 (63.9%)
Stage 3	7 (19.4%)
**Medical illness** (n, %)	
Human immunodeficiency viral illness	3 (8.3%)
**Cerebrospinal fluid opening pressure (lumbar puncture on admission), cm H2O(mean±SD)**	29.84±18.16 (range 1.5–75, median 29)
**Case definition of TBM** (n, %)	
Definite	23 (63.9%)
Probable	4 (11.1%)
Possible	9 (25%)

Hydrocephalus developed in 26 (72.2%) patients during the course of the illness and 14 (53.8%) of them required external ventricular drainage (EVD). Seven (26.9%) underwent ventriculoperitoneal shunt. Median lumbar CSF opening pressure was 29 cm H2O (range 1.5–75 cm H2O). Admission CT scan of the brain demonstrated new cerebral infarction in 8 (22.2%) patients. The PI of MCA was weakly correlated (r = 0.21) with lumbar CSF opening pressure (p = 0.31).

#### Findings in Phase I

2 (13.3%) patients in Phase I developed permanent hemiparesis.

#### Findings in Phase II

In our patients with Phase II, seven out of 11 patients (63.6%) had reduced Vmean with normal PI, and three patients had reduced Vmean with increased PI. Only one patient (9.1%) had reduced Vmean with reduced PI. Importantly, only one patient in phase II could be discharged from the hospital, but with permanent neurological deficits.

#### Findings in Phase III

Two (10.5%) patients progressed from phase I to phase III. The condition of 2 (5.3%) patients improved. One patient improved from phase III to phase II and the other patient from phase III to phase I. One of the four (25%) patients in phase III died.

The time course of Vmean and PI of the various intracranial arteries is illustrated in (S1 Table).

Briefly, 11 (30.6%) demonstrated stenotic velocities in the MCA, with 6 (16.7%) of them showing bilateral involvement. Eight (22.2%) patients had stenotic velocities in the ACA. Two (5.6%) patients had bilateral ACA involvement.

Table 2 lists the comparison of Vmean and PI between the patients who presented at an early stage (stage 1) and advanced stages (stages 2 and 3) on admission. There was a statistically significant difference when a comparison was made between early stage and advanced stages for Vmean of right PCA (p = 0.019) and basilar artery (p = 0.013). There was also a significant difference between early stage and advanced stages for Vmean of right ACA (p = 0.066). However, there was no statistically significant difference between the PI of the two groups.

**Table 2:** Mean flow velocities and pulsatility index in the patients with various stages of admission. ACA-anterior cerebral artery; BA- basilar artery; MCA-middle cerebral artery; PCA- posterior cerebral artery.

**MFV**	**Stage 1**	**Stage 2 and 3 (advanced stages)**	**P value**
L MCA (mean)	100.57±37.08	81.5±33.25	0.20
R MCA (mean)	104.71±33.52	84.63±43.78	0.27
MCA (Average of both MCA)	102.64±33.08	82.16±35.10	0.17
L ACA (mean)	68.86±17.65	61.08±17.65	0.46
R ACA (mean)	73±24.86	55.48±20.88	**0.066**
ACA (Average of both ACA)	71.5±20.03	58.7±19.67	0.14
L PCA (mean)	23.43±4.65	24±9.85	0.88
R PCA (mean)	33.43±18.34	22.64±6.7	***0*.*019***
PCA (Average of both PCA)	28.43±11.16	23.67±7.24	0.18
BA (mean)	62.43±11.32	45.17±16.13	***0*.*013***
**PI**	**Stage 1**	**Stage 2 and 3**	**P value**
L MCA (mean)	0.67±0.23	0.82±0.32	0.26
R MCA (mean)	0.72±0.23	0.85±0.33	0.35
MCA (Average of both MCA)	0.7±0.23	0.83±0.31	0.28
L ACA (mean)	0.7±0.22	0.77±0.43	0.71
R ACA (mean)	0.73±0.2	0.81±0.23	0.42
ACA (Average of both ACA)	0.72±0.2	0.81±0.28	0.41
L PCA (mean)	0.87±0.29	1.04±0.55	0.45
R PCA (mean)	0.97±0.53	1.01±0.47	0.82
PCA (Average of both PCA)	0.92±0.36	1.08±0.44	0.38
BA (mean)	0.75±0.14	0.9±0.3	0.22

Out of the 11 patients with stenotic velocities by TCD criteria, ten patients had Lindegaard ratio > 3 in the MCA. Seven patients (63.6%) had severe MCA vasculopathy (Lindegaard ratio > 6), which improved with time.

TBM vasculopathy with Lindegaard ratio > 3 in the MCA occurred as early as the fourth day of hospitalization and persisted up to four months. In addition, TBM vasculopathy with Lindegaard ratio > 3 in the MCA recurred in one patient. Two patients (18.2%) with TBM vasculopathy and Lindegaard ratio > 3 had more severe involvement in one MCA compared to the contralateral MCA.

### Imaging findings

Out of the 11 patients with stenotic velocities on TCD, one did not have either CTA or MRA. Eight out of the 10 patients (80%) with TBM vasculopathy based on TCD criteria, also had narrowing on CTA or MRA (Table 3).

**Table 3:** Patients who fulfilled the diagnostic criteria of MCA stenosis in both serial TCD and MRA/CTA studies and their cerebral infarcts.

	TCD	MRA/CTA	Cerebral infarcts
Patient 1	Bilateral MCA vasculopathy (Lindegaard ratio > 3)	Right MCA narrowing	Right parietal, right basal ganglia (right globus pallidus, right putamen)
Patient 2	Left MCA vasculopathy (Lindegaard ratio < 3)	Left MCA narrowing	Left temporal
Patient 3	Bilateral MCA vasculopathy (Lindegaard ratio > 3)	Both MCA narrowing	Both basal ganglia (left putamen, right caudate, right globus pallidus),both thalami, right parasagittal
Patient 4	Right MCA vasculopathy (Lindegaard ratio > 3)	Right MCA narrowing	Right basal ganglia (right globus pallidus)
Patient 5	Left MCA vasculopathy (Lindegaard ratio > 3)	Both MCA narrowing	Right basal ganglia (right putamen)
Patient 6	Bilateral MCA vasculopathy (Lindegaard ratio > 3)	Right MCA narrowing	Right basal ganglia (right caudate, right putamen)
Patient 7	Bilateral MCA vasculopathy (Lindegaard ratio > 3)	Left MCA narrowing	Both basal ganglia (both putamen, left caudate), left thalamus, left thalamus, right corpus callosum
Patient 8	Bilateral MCA vasculopathy (Lindegaard ratio > 3)	Left MCA narrowing	Right internal capsule, left corona radiata, right pons

### Functional outcome

Ten (27.8%) patients achieved good outcome (mRS 0–2) at 3 months, which increased to 13 (36.1%) at 6 months. The comparison of Vmean and PI between the patients who had functional outcome (mRS) at 3 and 6 months is shown in Tables 4 and 5. There was also a significant difference between good and poor outcome for Vmean of left MCA (p = 0.086) at 3 months, and Vmean of right MCA (p = 0.088) at 6 months. There was no statistically significant difference between the PI of both groups.

**Table 4:** Mean flow velocities and pulsatility index correlation with Modified Rankin scale (MRS) at 3 months. ACA-anterior cerebral artery; BA- basilar artery; MCA-middle cerebral artery; PCA- posterior cerebral artery.

**MFV**	**MRS 0–2 at 3 months**	**MRS 3–6 at 3 months**	**P value**
L MCA (mean)	101.1±27.61	78.89±35.31	**0.086**
R MCA (mean)	83.3±35.66	90.78±45.15	0.64
MCA (Average of both MCA)	92.2±29.7	83.88±37.28	0.53
L ACA (mean)	64.1±14.16	62.24±27.89	0.84
R ACA (mean)	53.6±20.81	61.38±23.24	0.37
ACA (Average of both ACA)	57.75±16.19	62.83±21.71	0.51
L PCA (mean)	24.2±4.71	23.7±10.43	0.89
R PCA (mean)	29.5±16.39	22.95±6.97	0.12
PCA (Average of both PCA)	26.85±9.51	23.74±7.68	0.33
BA (mean)	54.3±15.33	46.46±17.01	0.22
**PI**	**MRS 0–2 at 3 months**	**MRS 3–6 at 3 months**	**P value**
L MCA (mean)	0.73±0.16	0.81±0.35	0.52
R MCA (mean)	0.82±0.27	0.82±0.33	0.98
MCA (Average of both MCA)	0.78±0.2	0.82±0.33	0.72
L ACA (mean)	0.78±0.2	0.74±0.45	0.81
R ACA (mean)	0.89±0.19	0.79±0.24	0.82
ACA (Average of both ACA)	0.79±0.17	0.79±0.29	1.00
L PCA (mean)	0.96±0.24	1.02±0.54	0.53
R PCA (mean)	1.08±0.54	0.97±0.44	0.53
PCA (Average of both PCA)	1.02±0.35	1.06±0.46	0.81
BA (mean)	0.84±0.19	0.88±0.31	0.73

**Table 5:** Mean flow velocities and pulsatility index correlation with Modified Rankin scale (MRS) at 6 months. ACA-anterior cerebral artery; BA- basilar artery; MCA-middle cerebral artery; PCA- posterior cerebral artery.

**MFV**	**MRS 0–2 at 6 months**	**MRS 3–6 at 6 months**	**P value**
L MCA (mean)	89.73±37.11	71.75±38.75	0.62
R MCA (mean)	71.75±38.75	97.46±42.12	**0.088**
MCA (Average of both MCA)	78.29±37.28	78.29±37.28	0.34
L ACA (mean)	54.36±24.05	67.5±23.35	0.18
R ACA (mean)	51.45±20.76	61.23±17.6	0.23
ACA (Average of both ACA)	53.41±17.6	63.91±20.12	0.15
L PCA (mean)	24±4.99	24.21±10.49	0.95
R PCA (mean)	28.27±16.00	23.55±7.10	0.26
PCA (Average of both PCA)	25.86±9.53	24.43±7.73	0.65
BA (mean)	51.5±15.74	47.93±17.61	0.57
**PI**	**MRS 0–2 at 3 months**	**MRS 3–6 at 3 months**	**P value**
L MCA (mean)	0.77±0.15	0.79±0.36	0.85
R MCA (mean)	0.87±0.24	0. 79±0.	0.47
MCA (Average of both MCA)	0.82±0.18	0.79±0.35	0.76
L ACA (mean)	0.73±0.31	0.78.±0.43	0.73
R ACA (mean)	0.83±0.17	0.79±0.30	0.58
ACA (Average of both ACA)	0.82±0.16	0.79±0.30	0.77
L PCA (mean)	0.94±0.24	1.03±0.61	0.65
R PCA (mean)	1.09±0.53	0.95±0.46	0.44
PCA (Average of both PCA)	1.03±0.35	1.06±0.47	0.88
BA (mean)	0.88±0.21	0.87±0.32	0.90

## Discussion

Our study shows the feasibility of TCD in the diagnosis and monitoring of TBM-related vasculopathy in adult patients. To our knowledge, this is the first prospective study to demonstrate that TCD may have a potential role in the future management of TBM.

TCD provides accurate information on intracranial blood flow velocities and identifies areas of intracranial stenosis. [5, 13] TCD is a non-invasive and a relatively inexpensive test. [4, 13] TCD can be performed at the bed-side, making it a valuable tool in the critically ill and ventilated patients.

TCD has been used in the evaluation of intracranial arteries involved in TBM among children. [4] However, cerebral hemodynamic changes in adults may not be similar due presumably to a more mature autonomic nervous system.

TBM related vasculopathy is a dynamic disease and hence, it may reflect the natural course of the disease process as well as the response to various treatment strategies. Beyond a certain degree of hemodynamic compromise, TBM-related vasculopathy may lead to cerebral infarcts and permanent disabilities. Thus, early evaluation of cerebral hemodynamic changes can assist in the management of critically ill TBM patients. [13] In addition, TCD monitoring in TBM may guide the management of elevated intracranial pressure. [15]

Although TBM is a generalized process, TBM vasculopathy involved both MCAs in only 16.7% of the patients in our study. This is considerably lower compared than the case series in children with TBM, where up to 70% patients developed bilateral MCA vasculopathy. [5] Similarly, involvement of anterior cerebral artery (22.2% in our study) was not observed in previous studies in children.

Nearly all our patients who presented at Phase II had reduced Vmean with normal or increased PI. Only one patient (9.1%) had reduced Vmean with reduced PI. These findings are suggestive of a more diffuse vasculopathy. [16] This finding challenges the classification of TBM by Kilic et al. [4] According to them, a reduced Vmean with normal or increased PI would qualify for classification as Phase II TBM.

Kilic et al demonstrated a strong correlation between the three phases of TBM and GCS. [4] Our findings are consistent with a previous study on various types of central nervous system infection, which showed that Vmean in the MCA was higher in patients with GCS scores of 14 and 15. [12] Furthermore, consistent association of reduced Vmean and elevated PI with reduced GCS supports diffuse vasculopathy. [16]

In our study, we observed that a combination of Vmean and PI were neither predictive of the stage of disease nor the functional outcome. This is in contrast to previous studies on bacterial meningitis where Vmean correlated with the clinical outcome. [12, 13, 17, 18] Perhaps, the small number of patients was responsible for the absence of this observation in our study. Interestingly, we observed that 13.3% of our study patients in phase I TBM vasculopathy developed permanent hemiparesis. In contrast, no TBM patient diagnosed with phase I vasculopathy in the study by Kilic et al, had permanent neurological deficit. [4]

Previous studies reported cerebral vascular abnormalities in acute bacterial meningitis as short-lived and occurring within the first two to three weeks of infection. [13] The maximum hemodynamic abnormality was noted in the first 3 to 5 days. [13, 17] Our study showed that the hemodynamic abnormalities could last up to 4 months. The chronicity of TBM-related inflammation is well established, and this could contribute to the prolonged hemodynamic changes. [2, 4] Moreover, the prolonged duration of elevated cerebral blood flow velocities, suggests that stenosis due to vasculitis, instead of vasospasm, is a likely mechanism for cerebral ischemia, similar to a previous study. [5]

One of the strengths of our study is the confirmation of TCD abnormalities by CTA or MRA. Involvement of the proximal segments of the major branches of the circle of Willis in bacterial meningitis is responsible for the hemodynamic changes secondary to transient stenosis in proximal segments of the arteries. [17] Previous neuroimaging studies have demonstrated the involvement of intracranial arteries, focal perfusion defects and reversibility of these changes with treatment. [13, 17] Activation of white blood cells due to sepsis and focal spasm due to irritation of the arterial wall are believed to be responsible for the occlusion of cerebral blood vessels. [17, 19] Two (20%) patients with abnormal TCD findings failed to show the corresponding arterial narrowing on CTA or MRA in our study. Perhaps, the reason for elevated flow velocities was inflammatory hyperemia, which often occurs in the first week of the illness. [13]

Involvement of the intracranial arteries is considered to be one of the more important determinants of functional outcome in TBM. [13, 17, 20, 21] Higher Vmean with lower PI has been reported to be associated with good functional outcome in acute bacterial meningitis. [4] However, only 27.8% of our TBM patients achieved good outcome at 3 months, compared to 67% of acute bacterial meningitis patients. [13] Perhaps, a longer clinical course and higher intracranial pressure for a more prolonged period contributed to the higher incidence of cerebral ischemia. [12, 20, 21]

Certain limitations of our study need to be acknowledged. First, the study included a small number of subjects to draw definite conclusions. Second, patients were included at various stages of the disease process, which could have contributed to some of the variations observed in their cerebral hemodynamic assessment. Third, some other factors that could alter cerebral hemodynamics (especially arterial CO2 levels, body temperature, inflammatory biomarkers and intracranial pressure) were not monitored precisely in each patient. However, despite these limitations, our study still provides important information about the cerebral hemodynamic alterations in patients with TBM.

In conclusion, our study shows that a considerable proportion of patients with TBM develop haemodynamic disturbances due to focal vascular narrowing that could last many weeks and even lead to parenchymal ischemia. TCD is a reliable tool for the diagnosis and monitoring of these hemodynamic alterations in the natural course of the disease as well as assessing the response to various therapeutic strategies. Larger and well-planned studies are required to establish the role of TCD in the management of TBM and other CNS infections and to improve outcomes.

## Supporting Information

S1 Table Time course of mean cerebral blood flow velocity (Vmean) and pulsatility index (PI) of the various intracranial arteries for all the patients (overall average). (DOC)

S1 File STARD checklist. (DOC)
