# Supplementary material for: Role of Transcranial Doppler in the Evaluation of Vasculopathy in Tuberculous Meningitis
Source: PLoS One. 2016 Oct 10;11(10):e0164266. doi: 10.1371/journal.pone.0164266 (PMC5056701; doi:10.1371/journal.pone.0164266)
Supplement: S1 Table — (DOC) [file pone.0164266.s001.doc]

**S1 Table*:* Time course of mean cerebral blood flow velocity (Vmean) and pulsatility index (PI) of the various intracranial arteries for all the patients (overall average)**

|  | **Week 2** | **Week 4** | **3 months** | **6 months** |
| --- | --- | --- | --- | --- |
| **MCA** |  |  |  |  |
| Vmean | 91 | 97 | 75 | 65 |
| PI | 0.53 | 0.62 | 0.98 | 0.57 |
| **ACA** |  |  |  |  |
| Vmean | 43 | 62 | 62 | 62 |
| PI | 0.63 | 0.59 | 0.9 | 0.57 |
| **PCA** |  |  |  |  |
| Vmean | 29 | 23 | 20 | 25 |
| PI | 0.63 | 0.68 | 1.13 | 1.01 |
| **BA** |  |  |  |  |
| Vmean | 49 | 31 | 56 | 73 |
| PI | 0.75 | 0.53 | 0.9 | 0.53 |
